# BATF promotes tumor progression and association with FDG PET-derived parameters in colorectal cancer

**DOI:** 10.1186/s12967-024-05367-5

**Published:** 2024-06-11

**Authors:** Xia Lu, Jun Liu, Lijuan Feng, Yan Huang, Yanfeng Xu, Cuicui Li, Wei Wang, Yin Kan, Jigang Yang, Mingyu Zhang

**Affiliations:** 1 Department of Nuclear Medicine, Beijing Friendship Hospital, Capital Medical University, Beijing, 100050 China; 2 Department of Hepatobiliary and Pancreatic Surgery, Affiliated Hospital of Nantong University, Medical School of Nantong University, Nantong, 226001 China

**Keywords:** Colorectal cancer, BATF, Progression, ^18^F-FDG

## Abstract

**Purpose:**

The purpose of the study was to evaluate the expression and function of basic leucine zipper ATF-like transcription factor** (**BATF) in colorectal cancer (CRC), and its correlation with 2-deoxy-2[^18^F]fluoro-d-glucose (^18^F-FDG) positron emission tomography/computed tomography (PET/CT) parameters.

**Methods:**

The TIMER database, GEPIA database, TCGA, and GEO database were used to analyze the expression profile of BATF in human cancers. The reverse transcription‑quantitative PCR and western blot analyses were used to evaluate the mRNA level and protein expression in different CRC cell lines. The expression of BATF in SW620 and HCT116 cells was silenced and cell counting kit-8 assays and clonogenic assay were utilized to evaluate the role of BATF in CRC proliferation. The expression of tumor BATF and glucose transporter 1 (GLUT-1) were examined using immunohistochemical tools in 37 CRC patients undergoing preoperative ^18^F-FDG PET/CT imaging. The correlation between the PET/CT parameters and immunohistochemical result was evaluated.

**Results:**

In database, BATF was highly expressed in pan-cancer analyses, including CRC, and was associated with poor prognosis in CRC. In vitro, the results showed that knocking down of BATF expression could inhibit the proliferation of SW620 and HCT116 cells. In CRC patients, BATF expression was upregulated in tumor tissues compared with matched para-tumoral tissues, and was related with gender and Ki-67 levels. BATF expression was positively related to GLUT-1 expression and PET/CT parameters, including tumor size, maximum standard uptake value, metabolic tumor volume, and total lesion glycolysis. The multiple logistic analyses showed that SUV_max_ was an independent predictor of BATF expression. With 15.96 g/cm^3^ as the cutoff, sensitivity was 85.71%, specificity 82.61%, and area-under-the-curve 0.854.

**Conclusion:**

BATF may be an oncogene associated with ^18^F-FDG PET/CT parameters in CRC. SUV_max_ may be an independent predictor of BATF expression.

## Introduction

Colorectal cancer (CRC) is the third common neoplasms in terms of incidence, and the second in terms of mortality worldwide, with an estimated 1.9 million new cases and 935,000 deaths in 2020, representing about one in every 10 cancer cases and deaths [1]. Patients with local resectable tumor have a 5-year survival rate of 71%, while those with distant metastasis have a 5-year survival rate of approximately 13% [2]. Treatments include tumor surgery for the early-stage patients, with or without neoadjuvant chemoradiotherapy, followed by chemo- and/or radiotherapy for the advanced-stage patients [3]. Meanwhile, targeted therapies have shown promising benefits in several tumor types, such as breast cancer and melanoma [4, 5]. However, translating targeted therapies to the adjuvant setting of CRC remains a challenge [6]. Therefore, exploring the underlying pathogenesis and finding novel potential therapeutic targets for CRC are needed.

The basic leucine zipper ATF-like transcription factor family members (BATFs), including three members (BATF, BATF2 and BATF3), belongs to the activator protein 1 (AP-1) family of transcription factors [7]. BATF is also known as BATF1. BATFs were thought to function only as inhibitors of AP-1-driven transcription, but recent studies have highlighted their positive regulatory functions [8]. Transcription factor AP-1 regulates multiple genes and is involved in diverse cellular processes, including survival, differentiation, apoptosis, and development [9]. Several studies have reported the regulatory role of BATFs in solid tumors and hematologic cancers [10–13]. BATF2 is reported to inhibit epithelial-mesenchymal transition in CRC cells by downregulating transforming growth factor beta (TGF-β) [14]. BATF3 is reported to promote CRC cell proliferation and survival by forming AP-1 complexes with c-Jun in the cyclin D1 promoter, which controls cyclin D1 expression in CRC cell lines [15]. Thus, BATFs are considered as attractive targets for therapy and as prognostic indicators in CRC. As for BATF, it plays an important role in regulating differentiation and function in many lymphocyte lineages, such as T cells, B cells and dendritic cells [16]. However, the functional role of BATF in CRC is poorly understood. It was hypothesized that BATF acted as an oncogene in CRC.

The expression of BATF can be detected by immunohistochemistry (IHC) method. However, the constraint of IHC is the invasive surgery for detection, which is unable to repeatedly and dynamically monitor the expression level of BATF. As the heterogeneity of the tumor, the expression of BATF may be discordant between primary tumors and metastases, which may lead to false-negative results. As a molecular imaging modality, ^18^F-fudeoxyglucose positron emission tomography/computed tomography (^18^F-FDG PET/CT) is a non-invasive, non-traumatic, quantitative, real-time, and repeatable whole-body imaging technology, used for staging, re-staging, and assessing the treatment response in CRC cases [17]. The Semi-quantitative parameters of maximal standardized uptake values (SUV_max_) can reflect glucose metabolism in tumors, and are the most widely used PET/CT features, considered as an important indicator reflecting tumor aggressive biological behavior and rapid tumor proliferation [18]. The metabolic tumor volume (MTV) and total lesion glycolysis (TLG) theoretically integrate tumor volume and glucose metabolism, and can also reflect the metabolic activity of tumor cells, cell proliferation, and invasiveness [19]. However, the correlation between ^18^F-FDG PET/CT and the status of BATF has not been elucidated. As FDG uptake is affected by glucose transporters, and glucose transporter 1 (GLUT-1) expression has been revealed to be correlated with FDG uptake [20], the relationship among GLUT-1 expression, BATF expression, and FDG uptake was also investigated to explore the potential mechanism.

Therefore, this study aimed to investigate the oncogenic role and prognostic value of the BATF using public databases, CRC cell lines and human samples. The correlation between BATF expression and ^18^F-FDG PET/CT parameters was also investigated.

## Method

### BATF expression in databases

For human pan-cancer analysis, the Tumor Immune Estimation Resource (TIMER, https://cistrome.shinyapps.io/timer/) and the Gene Expression Profiling Interactive Analysis (GEPIA) database (http://gepia2.cancer-pku.cn/) were used to evaluate the expression of BATF in 33 enrolled cancer types, including colon cancer and rectal cancer [21, 22]. The mRNA expression levels of BATF in CRC and matched adjacent mucosa from Colon Adenocarcinoma and Rectum Adenocarcinoma projects of the Cancer Genome Atlas Program (TCGA-COADREAD) and the profile from Gene Expression Omnibus database (GEO-GSE87211), were compared using the two-tailed t test.

The CRC patients were obtained from the TCGA-COADREAD database, who were stratified into two groups based on BATF expression levels using the median value (50%) as the cut-off point, while the corresponding survival information was obtained from UCSC Xena database (http://genome.ucsc.edu) [23]. Then the value of BATF expression in the diagnosis of CRC, and in the prediction of overall survival (OS) were evaluated.

### Patients and tissues selection

A total of 37 patients who underwent ^18^F-FDG PET/CT imaging prior to surgical resection at our hospital from September 2016 to August 2022 were retrospectively included. Inclusion criteria were as follows: (a) CRC confirmed by pathology; (b) no biopsy, neoadjuvant radiotherapy or chemotherapy before PET/CT scan; (c) surgery performed within 2 weeks after PET/CT scan; (d) tissue samples available for IHC staining; and (e) complete clinical and pathological data. This retrospective observational study was approved by our institutional review board (Ethical approval number: 2023-P2-013-01). The use of the CRC tissues resected from patients who had undergone surgery at our institution was approved, and the requirement to obtain written informed consent was waived.

### Acquisition and analysis of ^18^F-FDG PET/CT imaging

In accordance with our institution’s PET/CT protocol [24], patients fasted for at least 6 h, with blood glucose levels less than 11.1 mmol/L prior to the intravenous administration of ^18^F-FDG (approximately 4.4 MBq/kg). All PET/CT examinations were performed using a Biograph mCT S64 (Siemens Healthineers Medical Solutions). The PET images were reconstructed using the ordered subset expectation maximization iterative method with CT data for attenuation correction.

The PET/CT images were independently reviewed by two experienced nuclear medicine physicians with more than 5 years of experience in PET/CT diagnosis, who were masked to the medical history of the participants. Any disagreement would be discussed till an agreement was reached. Tumor size, defined as the longest diameter of tumor, was obtained on a dedicated workstation (syngo MultiModality Workplace, Siemens). The semi-quantitative metabolic parameters, including SUV_max_, SUV_mean_, MTV and TLG were analyzed by two experienced nuclear medicine physicians using 3D slicer, a free open-source platform for medical image computing. Briefly, the volume of interests of the primary tumor was delineated using 3D slicer, then the SUV_max_ and SUV_mean_ were calculated on the PET image. A SUV_max_ threshold of 2.5 was used to define the MTV [25]. TLG was obtained as the product of SUVmean and MTV (TLG = MTV × SUV_mean_).

### Immunohistochemical staining and analysis

Tissue samples from the 37 CRC patients were trimmed and separately fixed with 10% paraformaldehyde, embedded in paraffin blocks and made into 4 µm-thick sections for immunohistochemical staining. After routine steps, one section was incubated with anti-BATF (Solarbio, K010096P, Rabbit), and another was incubated with anti-GLUT1 (MXB Biotechnologies, MAB-0813, mouse) at 4 ℃ overnight. Followed by poly-horseradish peroxidase anti-mouse/rabbit IgG detection system (ZSGB-BIO, PV-9000) as secondary antibodies at room temperature.

The results were scored independently by two experienced pathologists who were blinded to the patients’ information. BATF and GLUT-1 expression were comprehensively assessed in cross-sectional areas throughout the tumor and adjacent para-tumor tissues. BATF presented in the nucleus cytoplasm, while GLUT-1 localized primarily at the cell surface. The semi-quantitative BATF IHC score was determined by assigning the percentage of positive cells and staining intensity under a light microscope at a magnification of ×100. The percentage of positive-staining cells were scored as follows: 0% scored 0, 1–4% scored 1, 5–9% scored 2, 10–24% scored 3, 25–49% scored 4 and more than 49% scored 5. The staining intensity of BATF was graded as: no staining scored 0; weakly staining scored 1, moderately staining scored 2 and strongly staining scored 3. The final staining score was determined by combining the percentage scores and staining intensities. BATF low expression was defined as scores of 0 to 6 were, while high expression was defined as scores of 7–15. The GLUT-1 IHC score were calculated similarly to BATF, with 0%, 1–5%, 6–10%, 11–50%, 51–75%, and > 75% of positive-staining cells scored as 0, 1, 2, 3, 4, 5, respectively.

### Cell culture and reagents

The human CRC cell lines HCT8, HT29, SW480, HCT116, SW620, and LoVo were purchased from the Cell Bank of Type Culture Collection of the Chinese Academy of Sciences. SW620 and HCT116 were cultured in RPMI‑1640 medium (Gibco Corporation, USA). LoVo, HT29 and SW480 were cultured in DMEM (Gibco Corporation, USA). HT29 was cultured in McCoy's 5A (Gibco Corporation, USA).

All culture media were supplemented with 10% fetal bovine serum (Biological Industries, Israel) and 1% penicillin–streptomycin (Beyotime, China), and maintained at 37 °C with a humidified atmosphere of 5% CO_2_.

The BATF expression of the 6 CRC cell lines were detected by reverse transcription‑quantitative PCR (RT‑qPCR) in triplicate and Western blot analysis in once. And we selected the cell lines with the first and second highest BATF expression for the follow experiments.

### RT‑qPCR

Total RNA was isolated from CRC cells using RNAiso Plus (TAKARA, 9108, China). cDNA was synthesized using HiFiScript cDNA Synthesis Kit (CWBio, CW2569, China) following the instructions of manufacturer. The quantitation of mRNA expression of BATF was performed in triplicate by real time PCR (ABI PRISM 7500; Applied Biosystems, USA). The relative mRNA expression was normalized to the level of GAPDH and quantified by using the 2^−ΔΔCt^ method. The primer sequences were as follows:

BATF forward, TATTGCCGCCCAGAAGAGC;

BATF reverse, GCTTGATCTCCTTGCGTAGAG;

BATF2 forward, GCTGAAGAAGCAGAAGAACCGG;

BATF2 reverse, TGCAGGGACTGGATCTCCTTCC;

BATF3 forward, ACCGAGTTGCTGCTCAGAGAAG;

BATF3 reverse, AGGTGCTTCAGCTCCTCTGTCA;

GAPDH forward, GCACCGTCAAGGCTGAGAAC;

GAPDH reverse, TGGTGAAGACGCCAGTGGA.

### Western blot analysis

CRC cells were lysed in RIPA buffer (Beyotime, China) to extract protein. Protein concentrations were measured using a bicinchoninic acid protein assay kit (Beyotime, China). The lysate was stored at − 20 °C until further experimentation. Equivalent amounts of total protein were separated by SDS-PAGE gels and subsequently transferred onto polyvinylidene fluoride membranes (Millipore, USA). The membranes were blocked with 5% non-fat milk powder for 2 h at room temperature and incubated with the following primary antibodies over night at 4 °C: anti-BATF (Abcam, Ab236876, mouse, 1:1000), anti-BATF2 (ProteinTech Group, 16592-1-AP, mouse, 1:1000), anti-BATF3 (ABclonal, A14906, mouse,1:1000), and anti-GAPDH (Proteintech, 60004-1-Ig, mouse, 1:100000). The next day, the membranes were washed with TBST buffer three times and incubated with the corresponding horseradish peroxidase-conjugated secondary antibodies (Jackson, AB_10015289, goat anti-mouse, 1:5000) for 2 h at room temperature. Finally, the bands were detected by an electro chemiluminescence (ECL) detection kit (Millipore, USA) and visualized using a chemiluminescence imaging system (BLT, GV 6000M2, China). The relative expression of target proteins was measured by optical density of protein bands normalized to GAPDH.

### Vector construction and lentivirus transfection

Short hairpin RNA (shRNA) against BATF gene was designed using Broad Institute's GPP Web Portal and was constructed by using pTSB-U6-shRNA-EF1-copGFP-2A-PURO (Suzhou Yiji Biotechnology Co., Ltd.). Specificity was assessed using the NCBI BLAST tool before transfection. The shRNA was transfected into SW620 and HCT116 cells, while non-specific scrambled shRNA was used as the negative control (NC). The shRNA target sequences used for knockdown of BATF were sh1 CCGG-GGACTCATCTGATGATGTG-CTCGAG-CACATCATCAGATGAGTCC-TTTTTT, sh2 CCGG-ATGCAGAAGAGTATTAAGAAA-CTCGAG-TTTCTTAATACTCTTCTGCAT-TTTTTT, and sh3 CCGG-ACTCATCTGATGATGTGAGAA-CTCGAG-TTCTCACATCATCAGATGAGT-TTTTTT. To establish stable cell lines, 2 µg/ml puromycin was added to the medium every week following transfection with the lentiviral vectors. RT‑qPCR and western blot analysis were used in triplicate to verify the knockdown efficiency and specificity of the shRNA to BATF gene, not affecting other BATFs (BATF2, 3). The proliferation of cells was visualized under a fluorescence microscope (OPTIKA N-400LD, Italy).

### Cell proliferation assay

Cell counting kit-8 (CCK-8) was used to assess cell proliferation. Cells were inoculated in 96-well plates with 2000 cells/well, and each group had five replicate wells. At indicated time points, cells were cultured with 10 µl of CCK-8 solution (Beyotime, China) reagent for 2 h, and optical density at 450 nm of each sample was measured by Spectramax M3 (Molecular devices, USA) for plotting the viability curves.

### Clonogenic assay

The tumor cells were inoculated into six-well plates (1000 cells/well) in triplicate cultured for 7–14 days. When the colonies were visible, and each colony contained more than 50 cells under a microscope, the culture liquid was discarded. After fixation with paraformaldehyde solution (Servicebio, China) for 15 min, the plate was stained with crystal violet (Beyotime, China) for visualization. The number of colonies was counted with Image J (version 1.53C, USA).

### Statistical analysis

The R software (version 3.6.3) and corresponding R packages were used for data acquisition and statistical analyses for the data from databases. The SPSS 26.0 statistical package software (SPSS, Chicago, IL, USA) was used for the other statistical analyses. Figures were generated using GraphPad Prism 8 (GraphPad Software, San Diego, CA) and Python version 3.7.0 (Python Software Foundation, www.python.org). Continuous variables with a normal or skewed distribution were reported as mean ± standard deviation, median (interquartile range), respectively. And categorical variables were reported as numbers (percentages [%]). The distribution of continuous variables data was examined with the Shapiro–wilk test. Continuous variables were compared with the two-tailed independent t-test, Mann–Whitney, ANOVA, and Kruskal–Wallis tests. Categorical variables were compared with the two-tailed Fisher’s exact test. Correlation analyses were carried out using Spearman's correlation coefficient. The optimal cutoff value for receiver operating characteristic (ROC) curve was determined using the Youden index. Two-tailed multivariate logistic regression analysis was used to identify the primary predictor of BATF expression. The tolerance and variance inflation factor (VIF) were used to assess for multicollinearity among the independent variables. The OS was evaluated by Kaplan–Meier survival curve and the two-tailed Log-rank tests. For all statistical tests, two-tailed tests were used to determine statistical significance at *P*< 0.05.

## Results

### BATF is upregulated in pan-cancer and associated with poor prognosis in CRC from databases

**Fig. 1:**
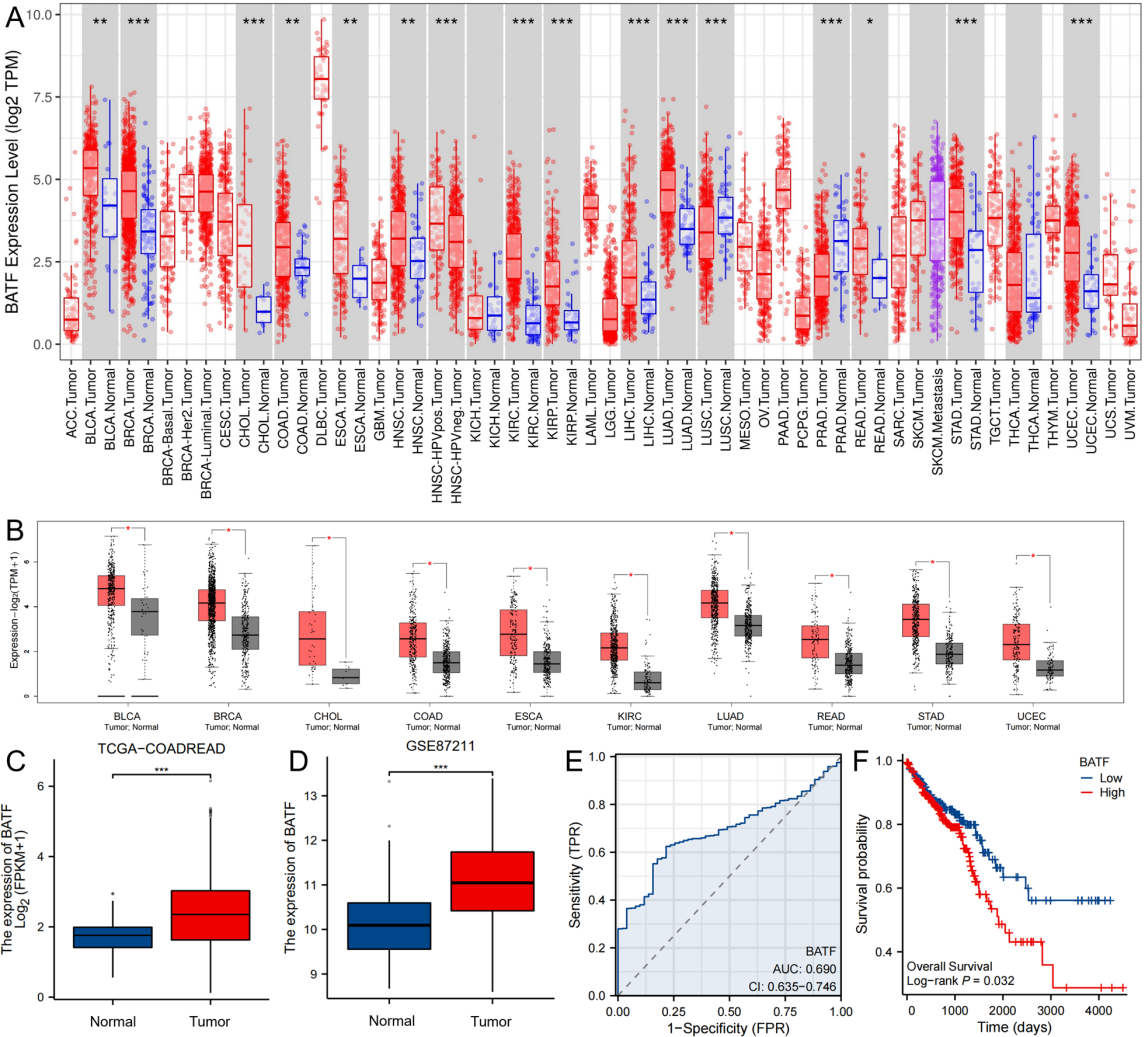
Up-regulation of BATF in pan-cancer and predicts prognosis of CRC in databases. **A**, **B** BATF expression in different tumor types in TIMER and GEPIA database. **C**, **D** Levels of BATF expression in CRC samples and normal samples in TCGA-COADREAD and GEO-GSE87211 databases. **E** The ROC curve of BATF expression in diagnosing CRC. **F** The K-M survival curves of high and low BATF expression groups. CRC: Colorectal cancer; BATF: Basic leucine zipper ATF-like transcription factor; ACC: Adrenocortical carcinoma; BLCA: Bladder urothelial carcinoma; BRCA, Breast invasive carcinoma; CESC: Cervical squamous cell carcinoma and endocervical adenocarcinoma; CHOL: Cholangio carcinoma; COAD: Colon adenocarcinoma; DLBC: Lymphoid Neoplasm Diffuse Large B-cell Lymphoma; ESCA: Esophageal carcinoma; GBM: Glioblastoma multiforme; HNSC: Head and Neck squamous cell carcinoma; KICH: Kidney chromophobe; KIRC: Kidney renal clear cell carcinoma; KIRP: Kidney renal papillary cell carcinoma; LAML: Acute Myeloid Leukemia; LGG: Brain Lower Grade Glioma; LIHC: Liver hepatocellular carcinoma; LUAD: Lung adenocarcinoma; LUSC: Lung squamous cell carcinoma; MESO: Mesothelioma; OV: Ovarian serous cystadenocarcinoma; PAAD: Pancreatic adenocarcinoma; PCPG: Pheochromocytoma and Paraganglioma; PRAD: Prostate adenocarcinoma; READ: Rectum adenocarcinoma; SARC: Sarcoma; SKCM: Skin Cutaneous Melanoma; STAD: Stomach adenocarcinoma; TGCT: Testicular Germ Cell Tumors; THCA: Thyroid carcinoma; THYM: Thymoma; UCEC: Uterine Corpus Endometrial Carcinoma; UCS: Uterine Carcinosarcoma; and UVM: Uveal Melanoma; *P < 0.05; **P < 0.01; ***P < 0.001

Based on the TIMER and GEPIA databases, we found that BATF was upregulated in various malignancies in both databases, including the bladder urothelial carcinoma, breast invasive carcinoma, cholangio carcinoma, colon adenocarcinoma (COAD), esophageal carcinoma, kidney renal clear cell carcinoma, lung adenocarcinoma, rectum adenocarcinoma (READ), stomach adenocarcinoma, and uterine Corpus Endometrial Carcinoma (Fig. 1A, B).

In the TCGA-COADREAD and GEO-GSE87211 databases, we included 644 and 51 cases of CRC, and 203 and 160 cases of matched adjacent tissues, respectively. The expression level of BATF in CRC tumors was higher than in the matched adjacent tissues (Fig. 1C, D). Based on the TCGA-COADREAD database, the ROC curve suggested the diagnostic potentials of BATF expression in CRC (AUC = 0.690, Fig. 1E). The K-M curves and log-rank test showed that high expression of BATF was correlated with worse OS (*P* = 0.032, Fig. 1F).

### BATF is highly expressed and promotes proliferation in CRC cell lines

**Fig. 2:**
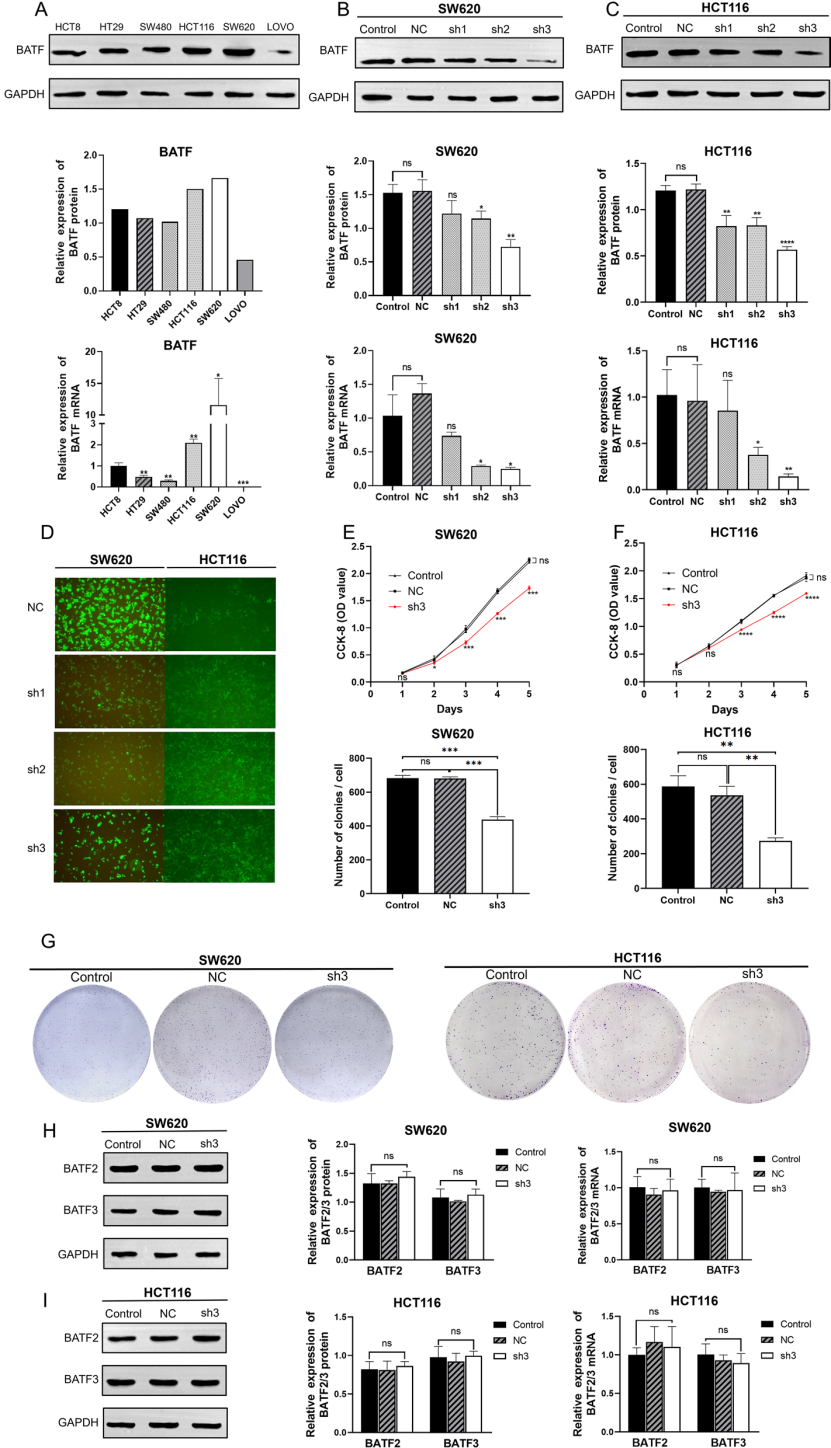
Silencing BTFA expression inhibited the proliferation of SW620 and HCT116 cells. **A** The western blot analysis and RT-qPCR of BATF expression in different CRC cell lines. **B**, **C** The western blot analysis and RT-qPCR confirming the success of BATF-shRNA transfection to SW620 and HCT116 cells. **D** The transfection efficiency of BATF-shRNA was observed under microscope. **E**–**G** CCK-8 assay and colony formation assay results showed the effects of BATF silencing on the proliferation ability of SW620 and HCT116 cells. **H**, **I** The western blot analysis and RT-qPCR confirming that the BATF-shRNA didn’t affect the expression of other BATFs (BATF2, 3). CRC: Colorectal cancer; BATF: Basic leucine zipper ATF-like transcription factor; shRNA: Short hairpin RNA; RT‑qPCR: Reverse transcription‑quantitative PCR; CCK-8: Cell counting kit-8; BATFs: BATF family members; *P < 0.05; **P < 0.01; ***P < 0.001

To explore the role of BATF in human CRC, we evaluated the BATF protein expression in six CRC cell lines. High expression of BATF was observed in HCT8, HT29, SW480, HCT116, and SW620 (Fig. 2A). We selected SW620 and HCT116 cell lines, with the first and second highest BATF expression, for subsequent experiments. Specific shRNA was used to silence BATF in SW620 and HCT116 cells, and our results of western blot and RT-qPCR assays revealed that sh3 had a good knockdown efficiency in both cell lines (Fig. 2B, C). A fluorescence microscope showed that the transfection efficiency was high (Fig. 2D).

CCK-8 assay and colony formation assay were performed to elucidate the function of BATF in cell proliferation. The results demonstrated that the proliferative and clonogenic ability of sh3 cells was slower than that of the Control and NC group in both SW620 and HCT116 cell lines (Fig. 2E–G).

Moreover, the results of western blot and RT-qPCR assays confirmed that the BATF-shRNA didn’t affect the expression of other BATFs (BATF2, 3) in both SW620 and HCT116 cell lines (Fig. 2H, I).

### BATF is highly expressed and correlated with clinicopathological features in CRC patients

**Fig. 3:**
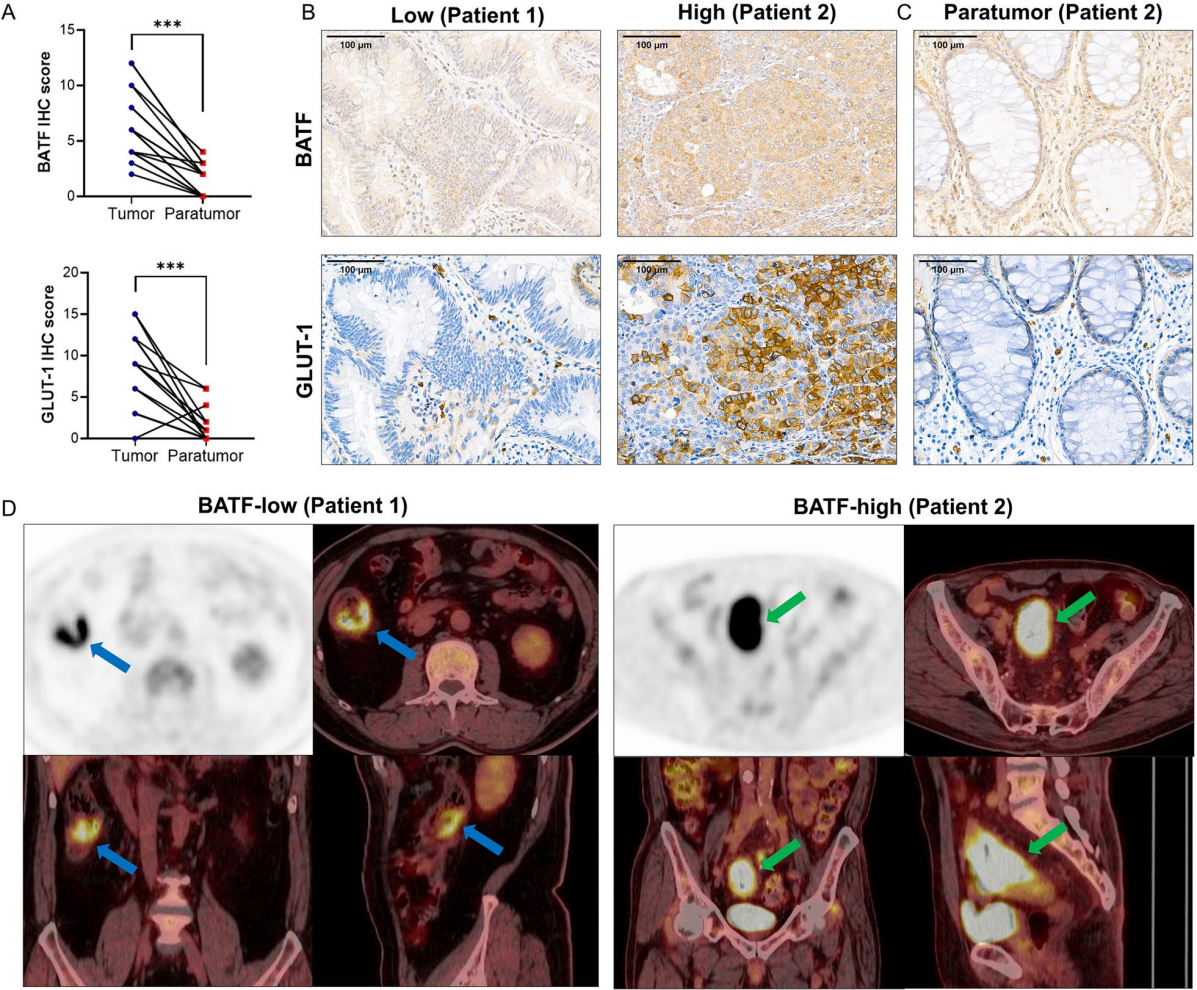
The expression of BATF and GLUT-1 in CRC patients. **A** The IHC score of BATF and GLUT-1 in the tumor tissues and matched paratumoral tissues from 17 patients. **B**–**D** Immunohistochemical staining results of BATF and GLUT-1 in tumor tissues and paratumoral tissues (× 400), and PET/CT imaging of patient 1and patient 2, with low and high expression of BATF, respectively. CRC: Colorectal cancer; BATF: Basic leucine zipper ATF-like transcription factor; GLUT-1: Glucose transporter 1; IHC: immunohistochemistry; PET/CT: Positron emission tomography/computed tomography; ***P < 0.001

The characteristics of 37 CRC patients and the relationship between BATF expression and various clinicopathological parameters are shown in Table 1. BATF was significantly highly expressed in CRC tumor tissues compared to matched paratumoral tissues from 17 patients (*P* < 0.0001, Fig. 3A–C). BATF expression was significantly higher in female than male, and in high Ki-67% levels than low (*P* = 0.039, 0.008, respectively). However, the results showed that the BATF expression was not associated with age, tumor location, tumor stage, differentiation, vascular invasion, lymphovascular invasion, lymph nodes metastases, carcinoembryonic antigen and carbohydrate antigen 19-9 levels in our study (*P* > 0.05).

**Table 1:** Clinicopathological characteristics of 37 CRC patients

Characteristics	All (n = 37)	BATF expression		*P* value
		Low (n = 23)	High (n = 14)	
Age, year	70 (57–77.5)	70.0 (61.0–78.0)	63.5 (54.0–77.0)	0.293
Gender, n (%)				0.039*
Male	24 (64.9)	18 (48.6)	6 (16.2)	
Female	13 (35.1)	5 (13.5)	8 (21.6)	
Tumor location, %				0.084
Right	12 (32.4)	10 (27.0)	2 (5.4)	
Left	25 (67.6)	13 (35.1)	12 (32.4)	
Stage, %				0.140
II	12 (32.4)	8 (21.6)	4 (10.8)	
III	8 (21.6)	7 (18.9)	1 (2.7)	
IV	17 (45.9)	8 (21.6)	9 (24.3)	
Differentiation, %				0.346
Well/Moderately	32 (86.5)	21 (56.8)	11 (29.7)	
Poorly	5 (13.5)	2 (5.4)	3 (8.1)	
Vascular invasion, %				0.493
Positive	15 (40.5)	8 (21.6)	7 (18.9)	
Negative	22 (59.5)	15 (40.5)	7 (18.9)	
Lymphovascular invasion, %				1.000
Positive	13 (35.1)	8 (21.6)	5 (13.5)	
Negative	24 (64.9)	15 (40.5)	19 (24.3)	
Lymph nodes, %				1.000
Positive	17 (45.9)	11 (29.7)	6 (16.2)	
Negative	20 (54.1)	12 (32.4)	8 (21.6)	
Ki-67, %				
≤ 50%	26 (70.3)	20 (54.1)	6 (16.2)	0.008**
> 50%	11 (29.7)	3 (8.1)	8 (21.6)	
Carcinoembryonic antigen	4.8 (2.6–17.6)	4.2 (2.3–10.9)	5.6 (3.6–22.4)	0.287
Carbohydrate antigen 19–9	19.6 (7.2–38.1)	20.7 (5.6–45.9)	18.5 (7.9–43.3)	1.000

CRC: Colorectal cancer; BATF: Basic leucine zipper ATF-like transcription factor; *P < 0.05; **P < 0.01

### BATF is related with GLUT-1 expression and PET/CT parameters in CRC patients

**Fig. 4:**
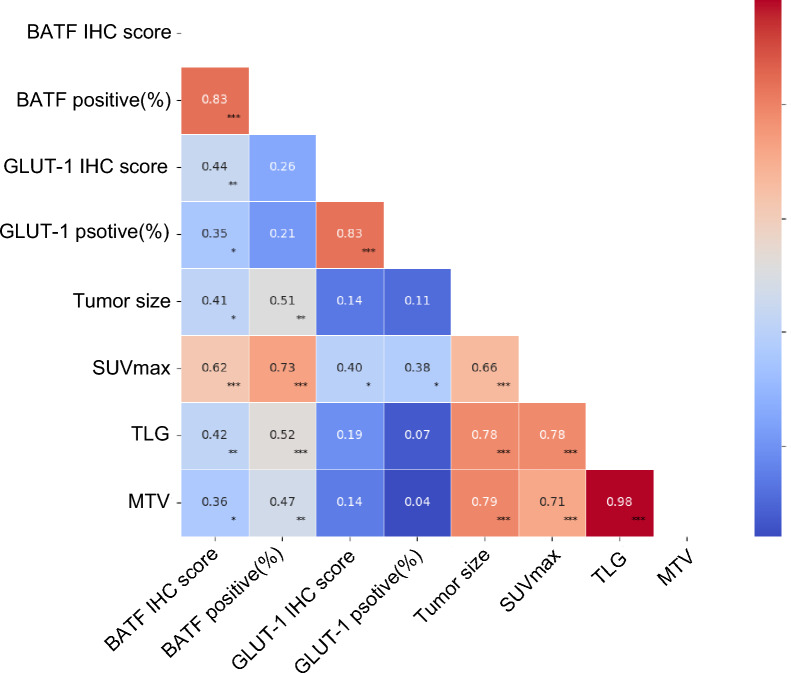
The correlation heatmap showed the correlation among BATF expression, GLUT-1 expression, and PET/CT parameters. CRC: Colorectal cancer; BATF: Basic leucine zipper ATF-like transcription factor; GLUT-1: Glucose transporter 1; IHC: immunohistochemistry; SUV_max_: maximal standardized uptake values; MTV: metabolic tumor volume; TLG: total lesion glycolysis; *P < 0.05; **P < 0.01; ***P < 0.001

**Fig. 5:**
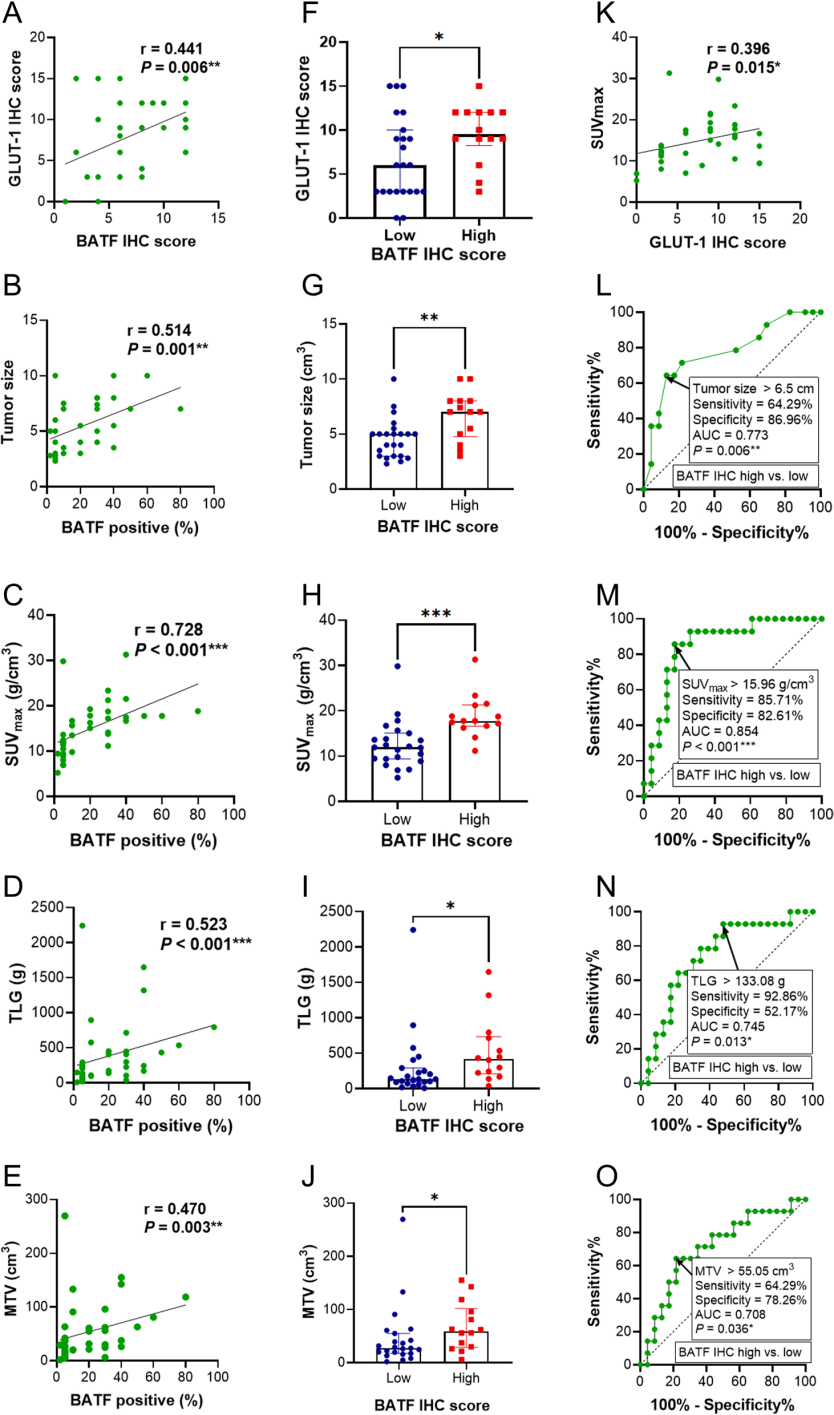
The correlation among BATF expression, GLUT-1 expression, and PET/CT parameters in CRC patients. **A**–**E** The scatter plots showed the correlation among BATF expression, GLUT-1 IHC score, tumor size, SUV_max_, TLG, and MTV. **F**–**J** The bar plots indicated the significant difference of GLUT-1 IHC score, tumor size, SUV_max_, TLG, and MTV in BATF IHC score low and high groups. **K** The scatter plots showed the correlation between GLUT-1 IHC score and SUV_max_. **L**–**O** The ROC curves represented the diagnosis efficiency of tumor size, SUV_max,_ TLG, and MTV to distinguish the high from low BATF IHC score groups. CRC: Colorectal cancer; BATF: Basic leucine zipper ATF-like transcription factor; GLUT-1: Glucose transporter 1; IHC: immunohistochemistry; SUV_max_: maximal standardized uptake values; MTV: metabolic tumor volume; TLG: total lesion glycolysis; *P < 0.05; **P < 0.01; ***P < 0.001

CRC patients were divided according to BATF expression: low and high expression groups (n = 21, 16, respectively). The IHC and PET/CT images of two typical patients (patient 1 and 2) with low and high expression of BATF are shown in Fig. 3B, D. Similar to BATF expression, GLUT-1 was also significantly highly expressed in CRC tumor tissues than matched paratumoral tissues from 17 patients (*P* < 0.0001, Fig. 3A–C). As shown in Figs. 4, 5A–E, our results demonstrated that BATF expression was positively correlated with GLUT-1 expression, tumor size, SUV_max_, TLG, and MTV (r = 0.441, 0.514, 0.728, 0.523, 0.470, respectively, *P* < 0.05). Moreover, the high-BATF expression group had higher GLUT-1 expression, bigger tumor size, higher SUV_max_, TLG, and MTV than the low-BATF group (*P* < 0.05, Figs. 4, 5F–J, Table 2). Furthermore, GLUT-1 expression was positively correlated with SUV_max_ (r = 0.396, *P* < 0.05, Fig. 5K). We determined the threshold of PET/CT parameters to distinguish patients with low and high BATF expression. The ROC curve analysis showed the cutoff value of tumor size, SUV_max_, TLG, and MTV were 6.5 cm, 15.96 g/cm^3^, 133.08 g, and 55.05 cm^3^, respectively (area-under-the-curve = 0.773, 0.854, 0.745, 0.708, respectively, *P* < 0.05, Fig. 5L–O).

**Table 2:** BATF expression is related with PET/CT parameters and GLUT-1 expression in CRC patients

18F-FDG PET/CT parameters	All (n = 37)	BATF expression		*P* value
		Low (n = 23)	High (n = 14)	
GLUT-1 IHC score	9.0 (3.0–12.0)	6.0 (3.0–10.0)	9.5 (8.3–12.0)	0.047*
Tumor size, cm	5.0 (3.5–7.0)	5.0 (3.0–5.0)	7.0 (4.8–8.0)	0.005**
SUV_max_, g/cm^3^	14.9 ± 14.1	11.9 (9.4–15.0)	17.7 (16.5–21.3)	< 0.001***
TLG, g	223.1 (103.8–447.0)	131.3 (88.6–290.6)	417.2 (209.2–732.8)	0.013*
MTV, cm^3^	36.3 (20.7–62.9)	27.1 (17.2–54.7)	58.8 (28.9–101.4)	0.036*

CRC: Colorectal cancer; BATF: Basic leucine zipper ATF-like transcription factor; GLUT-1: glucose transporter 1; IHC: immunohistochemistry; SUV_max_: maximal standardized uptake values; MTV: metabolic tumor volume; TLG: total lesion glycolysis; *P < 0.05; **P < 0.01; ***P < 0.001

### SUV_max_ is an independent predictor of BATF expression in CRC patients

Parameters with *P* < 0.05 were included in multivariate analysis. As multicollinearity among SUV_max_, TLG, and MTV (Tolerance < 0.1, VIF > 10) was observed, only SUV_max_ of the three parameters was included in multivariate logistic regression for predicting high BATF expression. Other three included parameters were gender, Ki-67% index, and tumor size. The results showed SUV_max_ was the only independent predictor of BATF expression in CRC in vivo (*P* = 0.008, Table 3), with the best diagnostic accuracy. With 15.96 g/cm^3^ as the cutoff, sensitivity was 85.71%, specificity 82.61%, and area-under-the-curve 0.854 (Fig. 4N).

**Table 3:** Multivariate analysis of BATF expression status in patients with CRC

Characteristic	OR	95%CI	*P* value
Gender male vs female	0.374	0.041–3.400	**0.382**
Ki-67% > vs ≤ 50%	3.548	0.381–33.067	**0.266**
Tumor size > vs ≤ 6.5 cm	3.408	0.328–35.418	**0.305**
SUV_max_ > vs ≤ 15.96 g/cm^3^	16.569	2.105–130.452	0.008**

CRC: Colorectal cancer; BATF: Basic leucine zipper ATF-like transcription factor; SUV_max_: maximal standardized uptake values; **P < 0.01

## Discussion

The global burden of CRC is expected to increase by 60% to more than 2.2 million new cases and 1.1 million deaths by 2030 [26]. Despite advances in cancer therapy, CRC with advanced stage remains a therapeutic challenge. Therefore, it is urgent to explore the mechanisms that regulate tumor pathogenesis and identify novel potential therapeutic targets. ^18^F-FDG PET/CT is a widely used staging modality of in various human neoplasms, especially for CRC. This study not only discussed the expression of BATF in CRC, but also identified its potential as an oncogene of CRC in vitro and in vivo, with a particular emphasis on the relationship between BATF expression and PET/CT parameters. Moreover, SUV_max_ was identified as an independent predictor of BATF expression, suggesting that ^18^F-FDG PET/CT could be a promising modality for diagnosis and treatment strategies in CRC.

BATF is a basic leucine zipper nuclear protein belonging to the AP-1 super-family of proteins [27]. Previous studies have shown that the BATF gene plays an important role in regulating differentiation and function in immune cells, such as T cells, B cells and dendritic cells [28]. Recent studies also showed that BATF plays an important role in the tumor microenvironment. Zhang et al. found that depletion of BATF in Chimeric antigen receptor T (CAR-T) cells enhances anti-tumor activity by inducing resistance against exhaustion and formation of central memory cells [29]. The study of Itahashi et al. found that BATF contributes to immunosuppression mediated by regulatory T cells [30].

BATF may acted as an oncogene in cancers. In our study, the human pan-cancer analysis showed the BATF is upregulated in many human cancers, including breast invasive carcinoma, kidney renal clear cell carcinoma, lung adenocarcinoma, colon and rectum adenocarcinoma, etc. These results are consistent with previous studies [10, 11, 31]. In our study, the RT‑qPCR and western blot analysis confirmed that BATF was upregulated in several primary CRC cell lines including HCT8, HT29, and SW480, as well as SW620 and HCT116. The IHC results also revealed that BATF was upregulated in CRC tumor tissues compared to matched adjacent tissues. These results suggested that BATF may play important roles in the progression of CRC in vitro and in vivo.

Moreover, studies also showed that BATF play important roles in the progression of tumor cells, promoting proliferation. In our study, high BATF expression correlated with higher Ki-67% index, reflecting the tumor cell proliferation. Our results of CCK-8 and clonogenic assay provides evidence that BATF knockdown significantly inhibited the proliferative and clonogenic ability of SW620 and HCT116 CRC cells, largely consistent with previously studies. Feng et al. found that knockdown of BATF inhibited the proliferation of A549 cells and promoted apoptosis in non‑small cell lung cancer [11]. Zhang et al. reported BATF promoted the proliferation of T47D and MCF-7 breast cancer cells [10]. The function of BATF in the migration and invasiveness of CRC was not studied in our research, as no correlation between BATF expression and vascular invasion, lymphovascular invasion and lymph nodes metastases was shown. It may be resulted by the small sample size, and the constraint of IHC, which means the BATF IHC results could not reflect the whole tumor status. However, a study reported that BATF promoted migration and invasiveness of breast cancer by facilitating the epithelial-mesenchymal transition via BATF/TGFβ1 axis, which positively regulated the expression of pro-metastatic proteins, CD147, MMP-2, and MMP-9 [10]. Therefore, further research with larger sample size and more experiments is needed to elucidate the role of BATF in promoting the migration and invasiveness in CRC.

The data presented in this study demonstrate that BATF could serve as a promising novel therapeutic target for CRC. Currently, there is a lack of commercially available inhibitors specifically targeting BATF. The findings of studies, however, demonstrated the potential of AP-1 inhibitors in suppressing BATF function. The compound T-5224 is a derivative of benzophenone, which acts as an inhibitor of the transcription factor c-Fos/activator protein (AP)-1. It exhibits anti-inflammatory and anti-cancer effects by specifically blocking the DNA binding activity of c-Fos/c-Jun to the AP-1-binding motif known as the TPA-responsive element (TRE) (5′-TGAGTCA-3′), thereby inhibiting C-FOS/C-JUN-mediated transcription [32]. A study on osteoarthritic, an inflammatory disease, demonstrated that T-5224 effectively inhibited the binding of BATF, JUN-B, and C-JUN proteins to TRE and significantly suppressed the upregulation of MMP3, MMP13, and ADAMTS5 induced by BATF in chondrocytes [33]. Additionally, a study conducted on head and neck squamous cell carcinoma demonstrated that T-5224 could effectively inhibit cancer invasion and migration in vitro, and had been has been confirmed to be safe in preventing lymph node metastasis in mice, with a daily dosage of 150 mg/kg [34].

The expression of BATF could be a potential selective criterion of anti-cancer therapeutics, which could only be detected after surgery and could not be monitored repeatedly and dynamically. As a whole-body molecular imaging modality, ^18^F-FDG PET/CT plays an important role in tumor diagnosis, staging and treatment outcome in CRC [35]. The PET-related parameters include the SUV_max_, MTV, and TLG, which were reported to be positively correlated with the malignancy of multiple tumors, including CRC [36]. Our study revealed that ^18^F-FDG PET/CT parameters could be useful non-invasive and repeatable methods to reflect the expression of BATF, and had the potential to guide the anti-cancer therapeutics of BATF inhibitors or AP-1 inhibitors prior CRC surgery. And SUV_max_ > 15.96 g/cm^3^ could be a useful cut-off value to predict high BATF expression.

Our study showed that GLUT-1 expression was positively correlated with SUV_max_, which is consistent with prior study [20]. Furthermore, our results furthermore demonstrated that the BATF expression was related with GLUT-1, suggesting that BATF might increase the uptake of ^18^F-FDG by up-regulating the expression of GLUT-1. It's well-known that TGF-β signaling is a critical regulator of glucose metabolism through regulation of GLUT-1 expression [37]. Previous studies have shown that TGF-β1 causes an increase in aerobic glycolysis in hepatic stellate cells and induces GLUT-1 expression by activating the Smad, p38 MAPK and P13K/AKT signaling pathways [38]. In CRC cells, it’s also reported that GLUT-1 gene plays an important role in the proliferation, differentiation, and apoptosis by regulating the TGF-β/PI3K-AKT-mTOR signaling pathway [39]. BATF may regulate the glucose metabolism of CRC via TGF-β and PI3K-AKT signaling pathway, and further studies are needed to elucidate the mechanism.

The major limitation of our study was the small sample size. Additionally, this retrospective study may have been affected by selection bias. Therefore, the present findings should be interpreted with caution. Moreover, more case series or prospective studies are needed to ratify the value of BATF expression and SUV_max_ in CRC.

## Conclusion

In conclusion, this study shows that BATF is highly expressed in CRC cell lines and tissues, and is correlated with the proliferative and clonogenic ability of CRC cells. Besides, BATF expression is positively correlated with ^18^F-FDG uptake, and SUV_max_ may serves as an independent predictor of BATF expression levels. These findings suggest that BATF may function as an oncogene in the progression of CRC, and that ^18^F-FDG PET/CT could offer a noninvasive means of predicting BATF expression status in CRC patients.

## Data Availability

The datasets used and/or analysed during the current study are available from the corresponding author on reasonable request.

## Abbreviations

CRC: Colorectal cancer
BATF: Basic leucine zipper ATF-like transcription factor
BATFs: BATF family members
^18^F-FDG: 2-Deoxy-2[^18^F]fluoro-d-glucose
PET/CT: Positron emission tomography/computed tomography
GLUT-1: Glucose transporter 1
IHC: Immunohistochemistry
SUV_max_: Maximal standardized uptake values
MTV: Metabolic tumor volume
AP-1: Activator protein 1
TLG: Total lesion glycolysis
TGF-β: Transforming growth factor beta
TIMER: Tumor Immune Estimation Resource
GEPIA: The Gene Expression Profiling Interactive Analysis
TCGA: The Cancer Genome Atlas Program
GEO: Gene Expression Omnibus database
COAD: Colon adenocarcinoma
READ: Rectum adenocarcinoma
OS: Overall survival
RT‑qPCR: Reverse transcription‑quantitative PCR
shRNA: Short hairpin RNA
NC: Negative control
CCK-8: Cell counting kit-8
ROC: Receiver operating characteristic
VIF: The tolerance and variance inflation factor
